# Editorial for the Special Issue “3D Printing of Gel-Based Materials (2nd Edition)”

**DOI:** 10.3390/gels12060513

**Published:** 2026-06-10

**Authors:** Aitor Tejo-Otero

**Affiliations:** 1 Department of Graphic Design and Engineering Projects, Faculty of Engineering in Bilbao, University of the Basque Country (UPV/EHU), 48013 Bilbao, Spain; aitor.tejo@ehu.eus; 2 BIOMAT Research Group, Escuela de Ingeniería de Gipuzkoa, University of the Basque Country (UPV/EHU), 20018 Donostia-San Sebastián, Spain

## 1. Introduction

The second edition of 3D Printing of Gel-Based Materials builds upon a previous volume, continuing to explore the rapidly advancing field of additive manufacturing with soft matter systems. While the first edition comprised nine research papers, this updated collection presents five carefully selected contributions, reflecting the most recent developments and focused advances in gel-based 3D printing technologies.

All the works included in this edition center on gels, which are generally defined as three-dimensional polymeric or colloidal networks capable of retaining a significant amount of liquid within their structure. These materials exhibit unique viscoelastic properties, combining solid-like and liquid-like behavior, making them particularly suitable for applications in bioprinting, soft robotics, pharmaceuticals, and food engineering.

This edition thus serves as a concise yet insightful update, offering readers a focused perspective on current trends and innovations in the field of gel-based 3D printing.

## 2. Papers Published in the Present Issue

The Special Issue “3D Printing of Gel-Based Materials (2nd Edition)” published in the journal *Gels* gathers five contributions that collectively reflect current advances in additive manufacturing of soft materials, particularly hydrogels, bioinks, and hybrid gel systems.

Two of the published papers are comprehensive review articles that provide broad perspectives on emerging materials and technologies. One review [contribution 1] focuses on tragacanth-gum-based bioinks, highlighting their potential as natural, biocompatible components for extrusion-based bioprinting. It discusses their rheological behavior, high water retention, and structural stability, emphasizing their suitability for tissue engineering applications and their role in improving printability and cell compatibility. Another review [contribution 2] explores stimuli-responsive hydrogels for 4D printing, analyzing how these smart materials react to environmental triggers such as temperature, pH, or light. This work connects material design, fabrication techniques, and applications, stressing the importance of programmable and adaptive systems for future applications in soft robotics, biomedical devices, and advanced manufacturing.

The remaining three contributions are research articles that address key experimental and technological challenges. One paper [contribution 3] presents a theranostic hydrogel fabricated via vat photopolymerization for advanced wound care. By integrating diagnostic sensing (through colorimetric pH response) and controlled drug delivery, the study demonstrates how 3D-printed gels can enable personalized and multifunctional medical treatments. Another article [contribution 4] develops a multi-material extrusion platform capable of combining thermoplastics and gel-based inks to fabricate hybrid scaffolds. This work highlights the importance of process calibration and rheological control to achieve accurate deposition and mechanically stable structures suitable for tissue engineering. And last but not least, a research contribution [contribution 5] that focuses on optimizing extrusion-based bioprinting parameters for sodium alginate-based hydrogels. Using data-driven approaches such as support vector regression and particle swarm optimization, the authors establish predictive models that significantly improve printing precision and reproducibility. This study addresses a critical limitation in bioprinting, where parameter uncertainty often affects structural fidelity.

## Data Availability

Not applicable.

